# Short LNA-modified oligonucleotide probes as efficient disruptors of DNA G-quadruplexes

**DOI:** 10.1093/nar/gkac569

**Published:** 2022-07-08

**Authors:** Souroprobho Chowdhury, Jiayi Wang, Sabrina Pia Nuccio, Hanbin Mao, Marco Di Antonio

**Affiliations:** Imperial College London, Chemistry Department, Molecular Sciences Research Hub, 82 Wood Lane, London W12 0BZ, UK; Institute of Chemical Biology, Molecular Sciences Research Hub, 82 Wood Lane, London W12 0BZ, UK; Department of Chemistry and Biochemistry, Kent State University, Kent, OH 44242, USA; Imperial College London, Chemistry Department, Molecular Sciences Research Hub, 82 Wood Lane, London W12 0BZ, UK; Institute of Chemical Biology, Molecular Sciences Research Hub, 82 Wood Lane, London W12 0BZ, UK; The Francis Crick Institute, 1 Midland Road, London NW1 1AT, UK; Department of Chemistry and Biochemistry, Kent State University, Kent, OH 44242, USA; Imperial College London, Chemistry Department, Molecular Sciences Research Hub, 82 Wood Lane, London W12 0BZ, UK; Institute of Chemical Biology, Molecular Sciences Research Hub, 82 Wood Lane, London W12 0BZ, UK; The Francis Crick Institute, 1 Midland Road, London NW1 1AT, UK

## Abstract

G-quadruplexes (G4s) are well known non-canonical DNA secondary structures that can form in human cells. Most of the tools available to investigate G4-biology rely on small molecule ligands that stabilise these structures. However, the development of probes that disrupt G4s is equally important to study their biology. In this study, we investigated the disruption of G4s using Locked Nucleic Acids (LNA) as invader probes. We demonstrated that strategic positioning of LNA-modifications within short oligonucleotides (10 nts.) can significantly accelerate the rate of G4-disruption. Single-molecule experiments revealed that short LNA-probes can promote disruption of G4s with mechanical stability sufficient to stall polymerases. We corroborated this using a single-step extension assay, revealing that short LNA-probes can relieve replication dependent polymerase-stalling at G4 sites. We further demonstrated the potential of such LNA-based probes to study G4-biology in cells. By using a dual-luciferase assay, we found that short LNA probes can enhance the expression of c-KIT to levels similar to those observed when the c-KIT promoter is mutated to prevent the formation of the c-KIT1 G4. Collectively, our data suggest a potential use of rationally designed LNA-modified oligonucleotides as an accessible chemical-biology tool for disrupting individual G4s and interrogating their biological functions in cells.

## INTRODUCTION

**Figure 1.**
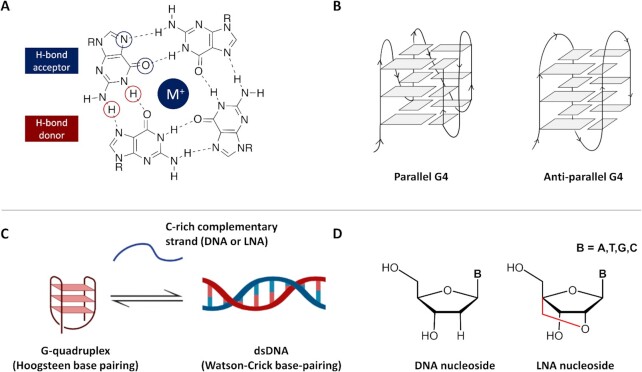
Illustration of a G4-structure and its potential disruption by duplex formation with its reverse complementary strand. (**A**) Schematic illustration of a G-tetrad, which is the core of a G4-structure. Four guanines interact in a tetrameric planar structure held together by Hoogsteen H-bonding, which can be further stabilised by alkali cations (M^+^), such as K^+^. (**B**) Stacking of three G-tetrads leads to the formation of a G4-structure, which can adopt either parallel or antiparallel topologies depending on the relative orientation of the four DNA strands in the G4-structure, as indicated by the arrows in the scheme. (**C**) G4-are formed from single-stranded DNA sequences. In the presence of their C-rich reverse complement, G4-folded sequences can be invaded to form a duplex DNA, which can be leveraged to disrupt the G4. (**D**) Chemical structures of DNA and LNA nucleosides. In LNA, an additional methylene (i.e. -CH_2_-) bridge shown in red ‘locks’ the nucleoside sugar conformation, significantly improving Watson–Crick H-bonding efficiency and DNA duplex stability.

G-quadruplexes (G4s) are non-canonical secondary structures of DNA that can form from guanine-rich sequences in human cells (1–4). Over the past two decades, chemical biology and bioinformatics approaches have unravelled more than half a million possible G4 structures in the human genome (5–7). Among these, >10 000 G4 structures have been shown to exist in the chromatinised DNA of pre-cancerous human keratinocyte cells (8). Single-molecule imaging of G4s in living cells under non-perturbative conditions has further confirmed that thousands of G4s can be detected in the nucleus of a living cell at any given time (9). Interestingly, promoters of transcriptionally active genes have been found to be particularly enriched in G4s, hinting towards a potential regulatory role of G4s in transcription (10). More recently, work by the Richter group has further corroborated these observations across different cell lines, which is consistent with the notion that promoter-G4s act as transcriptional regulators (11). Furthermore, several endogenous G4-unwinding helicases, such as Pif1, DDX5, XPBM and CSB, amongst others, have been reported to efficiently bind to or disrupt G4s, suggesting that the prevalence of G4s is tightly regulated in human cells (12–16). Although genomics approaches are becoming increasingly common for detecting G4s and gaining information about their biological roles, the use of small-molecule ligands that stabilise these structures remains one of the most direct ways to perturb G4-stability in real time and investigate biological phenotypes associated with G4s. Structurally, a G4 consists of four guanine bases coordinating through Hoogsteen hydrogen bonding to form planar structures known as G-tetrads (Figure 1A), which stack on top of each other to form G4s with topologies such as parallel or antiparallel (Figure 1B). Given that the planar G-tetrads are structurally fundamental to G4s, this structural feature can be exploited for the design of G4-binders. Indeed, much work has been done in the past 20 years during which more than a thousand small-molecules have been described to stabilise G4s through end-stacking interactions with the G-tetrads (17). However, mounting evidence has linked uncontrolled accumulation of G4s to diseases (18–21), highlighting that the development of probes that disrupt (rather than stabilise) G4s could be equally important to study the biological relevance of these DNA structures (12).

Currently molecules to disrupt G4s are limited, with only few molecular structures described to date (22–24). Recent work by the Monchaud group provided the first example of a chemical platform for rational identification of small-molecule ligands that disrupt G4s (25). However, small-molecule based G4-disrupting probes reported to date require significant synthetic chemistry efforts, making this approach less accessible to the wider biology community. Furthermore, small molecules are inherently unable to discriminate between different G4s, leading to genome-wide disruption of these structures that prevents investigations into the biology of an individual or a small subset of G4s. To overcome these limitations, G4-targeting approaches involving sequence-specific hybridisation of synthetic oligonucleotide probes have been proposed, particularly those exploiting the use of Peptide Nucleic Acid (PNA) constructs (26–28).

Although G4 stability *in vitro* is assessed using single stranded G-rich sequences, in the genome G4s are always exposed to their reverse-complementary strand (*i.e*. the C-rich strand) that competes with the G4 for duplex formation. These two structures (*i.e*. duplex and G4) are therefore likely to exist in a dynamic equilibrium (29) (Figure 1C), as recently demonstrated by single molecule live cell imaging (9). Inspired by this, Maiti and co-workers have identified Locked Nucleic Acids (LNA) (Figure 1D) as a promising nucleoside-modification for perturbing the equilibrium between G4s and dsDNA (30). More specifically, they have demonstrated that installing an increasing number of LNA-modifications in DNA oligonucleotides significantly increases the binding affinity of the sequences that are complementary to a given G4, which drives greater duplex formation (31). On the other hand, the addition of an excessive number of LNA-modifications in DNA oligonucleotides poses the risk of creating an LNA-modified duplex that is far too stable, which may perturb biological processes and would therefore not be suitable to investigate G4-biology. Nevertheless, LNA probes (32) offer distinct advantages over unmodified DNA and other nucleoside modifications such as PNA, including significantly improved mismatch discrimination (33) and water-solubility (34), which makes them ideal candidates for the strategic development of G4-disruptors.

To fully harness the potential of LNA-modifications to generate G4-disruptors, we systematically designed a panel of different LNA-modified sequences complementary to model G4 structures (c-KIT1 and hTelo) and investigated their ability to affect the kinetics of G4-disruption. We explored the effect of LNA-placement by designing sequences that differ in the positioning of the modifications along the probe, keeping the total number of LNA modifications under five. We observed that the LNAs placed opposite to guanines within a G-tetrad accelerated the rate of G4 disruption, whilst the very same sequences with the LNA targeting the loops of the G4 resulted either in non-significant changes in disruption kinetics or, counterintuitively, a deceleration of the G4-disruption. Strikingly, our investigations also revealed that an LNA-modified probe as short as 10 nucleotides retains the same G4-disrupting ability when compared to fully complementary sequences containing the same LNA modifications, suggesting that LNAs actively trigger the G4-disruption process and that short probes can be used to accelerate G4-disruption under physiological conditions. Single-molecule mechanical disruption experiments confirmed the enhanced G4-disruption by short LNAs, demonstrating the potential of such probes to deplete G4s sufficiently stable to stall polymerase. This led us to hypothesise that LNA-probes could facilitate the progression of polymerases through stable G4 sites across the genome during replication and transcription. We demonstrated this concept in a single-extension polymerase-stalling assay, revealing that treatment with LNA-probes can measurably enhance polymerase progression through G4-templates in a fashion that supports an LNA-mediated G4-disruption model. Remarkably, the probes with the greatest c-KIT1 G4-disrupting ability also caused an increase in c-KIT gene-expression, as measured by a dual luciferase assay. Altogether, our data demonstrates that short LNA probes can be easily designed to significantly disrupt G4-folding both in biochemical assays and in cells, suggesting a potential for rationally designed LNA probes as an accessible platform for systematic investigation of the biological function of individual G4s in the human genome.

## MATERIALS AND METHODS

### Bulk FRET G4 disruption kinetics

Oligonucleotides used in this assay were purchased from Integrated DNA Technology (IDT) and stored as water solutions at − 20°C (for a complete list of oligonucleotides used, see Supplementary Table S1). A 1 μM stock solution of 5′-FAM, 3′-TAMRA labelled c-KIT1 (5′-FAM/AGG GAG GGC GCT GGG AGG AGG GGC/TAMRA-3′) or hTelo (5′-FAM/GGG TTA GGG TTA GGG TTA GGG/TAMRA-3′) was prepared in a 10 mM Tris–HCl pH 7.4 buffer supplemented with either 1 mM KCl or 100 mM KCl and annealed by heating the solution at 95°C for 10 min, followed by cooling to room temperature overnight. Oligonucleotide solutions were stored in the dark at 4°C prior to the assay. Solutions were then diluted to obtain final labelled oligonucleotides concentration of 100 nM in volume of 100 μl. Disruption reactions were triggered by the addition of 500 nM complementary DNA or LNA sequence at time (*t*) = 0. Experiments were performed at 25°C. The samples were excited at 488 nm with an 8 nm excitation slit and emission was measured at 518 nm with an 8 nm emission slit at regular time points using a BMG Labtech Clariostar Plus instrument. Data points were taken every 30s (for hTelo) or every 120s (for c-KIT1). All experiments were performed in three independent replicates. Data were normalised to define the signal at *t* = 0 as 0% and the saturation point of the curves as 100%. For each condition, each replicate dataset was fit to a non-linear model using a single exponential decay function to obtain apparent half-lives (*s*). Data analysis and visualisation were performed using GraphPad Prism 9.0.1. Error bars represent standard error of mean (SEM). Statistical analysis was performed using an unpaired, two-tailed Student's t test and *P* values less than 0.05 were considered statistically significant.

### Optical tweezer measurements

The DNA oligomers were purchased from Integrated DNA Technology (IDT, Coralville, IA) and stored at −20°C. T4 DNA ligase and Exonucleases I and III were obtained from New England Biolab (NEB, Ipswich, MA). Terminal transferase and Phi 29 polymerase were respectively obtained from Thermo Fischer (Waltham, MA) and Mclab (South San Francisco, CA). Digoxigenin dUTP (Dig-dUTP) was purchased from Roche (Swiss). Streptavidin and digoxigenin (Dig) antibody-coated polystyrene beads were purchased from Spherotech (Lake Forest, IL).

### Preparation of ssDNA construct that contains multiple repeats of telomeric G-quadruplex (GQ) forming sequence

To synthesize the multi-GQ containing ssDNA construct used in single-molecule mechanical disruption experiments, 5′ phosphorylated DNA oligonucleotides (59 nt, 5′- GTC GTG ATG TAC CAT TCA GAA CTG TAA CCC TAA CCC TAA CCC TAA CCC TAA CAA TCC TG) that contain a 27-nt sequence (underlined) complementary to the hTelo DNA sequence was first slowly annealed (95–25°C in 2.5 h) with a splint (42 nt, 5′- GCA TTA GGA AGC AGC CCA GTA GTA GGA TCA CGA CCA GGA TTG). The hybridized DNA was circularized by T4 DNA ligase at 16°C for 16 h (Supplementary Figure S1A). After ligation, the splint was removed by a splint remover (42 nt, 5′- CAA TCC TGG TCG TGA TCC TAC TAC TGG GCT GCT TCC TAA TGC) which contains a complementary sequence to the splint. Exonuclease I and Exonuclease III (NEB) were then used to digest ssDNA and dsDNA at 37 °C for 12 h to remove all linear DNA strands in the product.

The circular template was then slowly annealed with a biotinylated primer (42 nt, 5′-biotin- GCA TTA GGA AGC AGC CCA GTA GTA GGA TCA CGA CCA GGA TTG) to carry out RCA (Rolling Circular Amplification) by Phi 29 polymerase in presence of 10 mM dNTP at 30 °C for 5 min. After RCA, the product was labeled with digoxigenin (Dig) at the 3′ end through the terminal transferase with 0.2 mM Dig-dUTP at 37 °C for 1.5 h. The biotin- and digoxigenin-labelled, multi-GQ containing ssDNA construct was stored at −20°C and used within one week for mechanical disruption experiments.

### Mechanical disruption of the multi-GQ containing ssDNA

A multi-channel microfluidic chamber was used for single-molecule experiments (Supplementary Figure S1B). The chamber was prepared by thermal bonding of a parafilm (thickness 0.127 mm, Bemis Co., Neenah, WI) between two pieces of cover glass (#1, VWR, size 24 × 60 mm). The parafilm with a microfluidic pattern and the cover glass with input and output holes was cut by a CO_2_ laser (Versa Laser, Scottsdale, AZ). The top and the bottom channels in the microfluidic chamber were used to inject streptavidin-coated polystyrene beads that had been attached with multi-GQ containing ssDNA and the anti-Digoxigenin-coated polystyrene beads, respectively. The top and bottom sections of the middle channel were connected with the top and bottom channels by micropipettes (Garner Glass Co., i.d. 25 μm), respectively. Buffers with different chemical compositions were flowed into the two middle channels by a Harvard Apparatus PHD 2000 pump (Harvard Apparatus, South Natick, MA) with a flow rate maintained at 0.5 μl/min. The chamber was fixed in a sample bracket in a homemade laser optical tweezer instrument (35). To start mechanical disruption experiments, an anti-Digoxigenin-coated polystyrene bead and a streptavidin-coated polystyrene bead attached with the multi-GQ containing ssDNA were trapped by two laser foci separately at 24°C. These two laser foci (350 mW power for each) were focused by an objective [Nikon CFI-PlanApochromat 60×, numerical aperture (NA) 1.2, water immersion, working distance ∼320 μm] from collimated S- and P-polarized laser beams, which were emitted from a CW diode-pumped solid-state laser (1064 nm, 5W, Spectrophysics). Using a steerable mirror (Nano MTA, Mad City Laboratories, Madison, WI), the DNA tethered, streptavidin-coated bead (1.87 μm diameter), was moved close to the anti-digoxigenin-coated bead (2.32 μm diameter) trapped by another laser. This action allowed the tethering of a single-molecule DNA construct between these two trapped beads due to affinity interactions. Once the tether was obtained, the ssDNA construct was extended by mechanical force at a 5.5 pN/s loading rate (in the range of 10–30 pN). The force in the DNA tether and the DNA extension between two optically trapped beads were recorded in a Labview 8.2 (National Instruments, Austin, TX) program and treated by Igor programs (Wavemetrics, Portland, OR) to generate force–extension curves. The mechanical disruption experiments were performed in a 10 mM Tris buffer (pH 7.4) containing 100 mM KCl with either 100 nM DNA probe or 100 nM LNA (Locked Nucleic Acids) probe. For calculation of the change in contour length (**Δ***L*) and construction of **Δ***L*-force curves, see Supporting Information, Supplementary Figures S2 and S3. The statistical analysis was performed by the unpaired, two-tailed Student's *t*-test on the mean of at least 5 independent measurements. Statistical significance is under *P* values lower than 0.05.

### Single-step polymerase extension assay

Polymerase extension assay was performed using Phi29 DNA polymerase (36) purchased from New England Biolabs (NEB). 10 μM of template DNA sequence (TCT GCT TTG GGA ACC CGA GAG GAG CGC TTA TAG GGA GGG CGC TGG GAG GAG GGA GGA GAC TCA GCC GAG CAG CCG AGC ACT CTA GCT CTA G) was annealed in presence of 1 μM of Cy5 labelled primer (5′-Cy5/CTA GAG CTA GAG TGC TCG GC-3′) to make up a final volume of 20 μl in either 100 mM KCl, 10 mM Tris–HCl pH 7.4 buffer or in 100 mM LiCl, 10 mM Tris–HCl pH 7.4 buffer. Oligonucleotides were annealed by heating the samples to 95°C for 6 min and then left at room temperature overnight to cool down. Separately, 1× Phi29 polymerase buffer (by diluting the 10× buffer in miliQ water), and 1× BSA (by diluting the manufacturer supplied 10× recombinant BSA in 1× Phi29 polymerase buffer) were prepared. The assay was performed in PCR tubes to have a final volume of 8 μl after mixing of all components required for the single-run extensions. Briefly, for achieving disruption of G4s, we mixed 100 mM KCl or LiCl (added: 0.4 μl from 2M stocks), 320 nM of the annealed template (added: 0.25 μl from 10 μM stock solution), and 1.3 μM of LNA or DNA (added: 0.1 μl from 100 μM stock solution) to 1× Phi29 polymerase buffer to a final volume of 7.2 μl, and the mixture was incubated at 25°C for 6 h. Successively, this mixture was added with 0.4 μl of 1× BSA, 0.2 μl of Phi29 Polymerase, and 0.20 μl of dNTPs (from 10 mM stock) up to a final volume of 8 μl. The PCR tubes were incubated at 30°C for 2.5 h and the reaction was terminated by adding 2 μl of 50% glycerol and 8 μl of a stop-solution (95% formamide, 10 mM EDTA, 10 mM NaOH), heated to 95°C for 10 min and flash-cooled on ice for another 15 min. 8 μl from each reaction was added to each well of a denaturing gel (15% PAGE TBE–urea, pre-run for 15 min at 300 V), and the gel was run for 55 min at 300 V. The imaging of the gels was performed using Typhoon FLA 9500 (GE Healthcare). The quantification of bands was performed using ImageJ. The assay was repeated as three independent experiments. Data analysis was performed on GraphPad Prism 9.0.1, where statistical hypothesis testing was performed using the unpaired, two-tailed Student's t-test. *P* values lower than 0.05 were considered statistically significant. Error bars in graphs represent standard error of mean (SEM).

### UV melting

UV-melting experiments were performed as previously described (37). In a typical experiment, equimolar amounts of c-KIT1 (5′-AGG GAG GGC GCT GGG AGG AGG GGC-3′) and complementary oligonucleotide (DNA or LNA, see Table 1) were mixed to a final concentration of 1 μM (200 μl) of duplex in 100 mM KCl Tris–HCl pH 7.4 buffer. Absorbance was monitored at 260 nm while heating samples from 25°C to 95°C, and subsequent cooling back to 25°C. The rate of heating/cooling was 0.8°C/ min with data interval of 0.1°C/ min, averaging time of 0.100 (s) and spectral bandwidth 2.00 nm. Consistent data were obtained through three independent repeats. For calculating melting temperature, the heating-profile from each independent experiment was normalised with the lower and higher asymptotes set at 0% and 100% respectively. The temperature corresponding to the 50% of the absorbance value from each experiment was used to extract *T*_m_. The triplicates were then used to generate mean curves and standard errors of means.

**Table 1.** List of oligonucleotide sequences used to target c-KIT1 G4. Bold with underline refers to LNA-modified nucleosides

Sequence ID	Sequence (5′ to 3′)
**KIT_LNA1**	CCC**TCCT**CCCAGCGCCCTCCC
**KIT_LNA2**	T**CCCT**CCTCCCAGCGCCCTCCC
**KIT_LNA3**	TC**C**CTCCTC**C**CAGCGC**C**CTC**C**C
**KIT_LNA4**	TCCC**T**C**C**TCCC**A**G**C**GCCCTCCC
**KIT_DNA1**	TCCCTCCTCCCAGCGCCCTCCC

### Dual luciferase assay

#### HEK293T cell culture

Cells were grown in DMEM Glutamax media (Gibco, 10566016) supplemented with 10% fetal bovine serum at 37°C, 5% CO_2_.

#### Luciferase assay

HEK293T cells were plated one day before transfection, at a cell density of 4 × 10^4^ cells/well in a 96-well plate (Corning^®^ 96-well Clear Flat Bottom-3598). The transfection was performed with 330 ng of pc-KIT plasmid (Addgene plasmid number 118983) and 25 μM of LNA probe in a final volume of 5 μl by using FuGENE® HD Transfection Reagent (Promega-E2311). The level of expression of the dual luciferase reporter genes was measured 24 h post-transfection. The luminescence signals were acquired using a BMG CLARIOSstar Plus luminometer, using the dual luciferase reporter assay reagents (Promega-E1980) and following the manufacturer instructions. Luminescence data were acquired from three biological replicates each performed in three technical replicates.

#### Statistical analysis for dual luciferase assay

The dataset containing the biological replicates for each condition was normalised to impose that the highest value in each the dataset was considered 100%. The normalised datasets (*N* = 3 for each condition) was tested using Shapiro-Wilk test (*P* > 0.05 in all cases, suggesting normal distribution) and Normal QQ-plots. The statistical significance of the results was tested using one-way ANOVA first to assess overall significant differences, and successively using the Dunett's test (mean of each condition was compared with the mean of control which is Plasmid-alone). *P* values lower than 0.05 were considered statistically significant. Error bars on graphs represent standard error of mean (SEM).

## RESULTS

### Design of LNA-modified G4 disrupting probes

To explore whether installing LNA-modifications along the reverse complementary strand of a G4 had a position-dependent effect on G4-disruption, we used, as our initial model system, the c-KIT1 sequence that forms an established parallel G4 with extensively characterised structure and disruption kinetics (9,38). We designed a set of five LNA-modified oligonucleotides that were reverse-complementary to the c-KIT1 G4, all of which contained a total of four LNA modifications but varied in their location within the sequence. To avoid excessive perturbations, we reasoned that four LNA modifications would cover ∼20% of the full length G4-sequence, which would confer substantial binding affinity to the probes based on prior biophysical knowledge on LNA-based probes (31). Using this strategy, we attempted to target various structural features of c-KIT1 G4, such as loops or G-tracts (Table 1).

To achieve this, **KIT_LNA1** was designed to have LNA modifications targeting the 3′ loop of the c-KIT1 G4; **KIT_LNA2** targets the 3′ G-tract; **KIT_LNA3** targets the middle guanine of each G-tract and **KIT_LNA4** has scrambled LNA modifications along the loops of the G4-sequence. Since the number of LNA modifications were kept constant in all the designs **KIT_LNA1-4**, it was expected that the stabilities of the final heteroduplexes formed upon annealing with c-KIT1 G4 would be similar. To investigate this, we performed 260 nm UV-melting of the DNA-LNA heteroduplexes formed between the c-KIT1 G4-folding sequence and each of the fully complementary LNA probes (**KIT_LNA1-4**). As expected, all the LNA probes caused a significant, but comparable, increase in the melting temperature of the duplex formed (∼8°C, see Supplementary Table S2 for Δ*T*_m_ values) compared to the duplex obtained using a non-LNA modified DNA probe. This is in line with previous literature reports, which confirms that the positioning of the LNA probes along the sequence has a limited effect on the thermodynamic stability of the duplex formed as a result of G4-disruption (39).

### Kinetics of c-KIT1 G4 disruption upon treatment with LNA-probes reveal a strong dependence on LNA positioning

**Figure 2.**
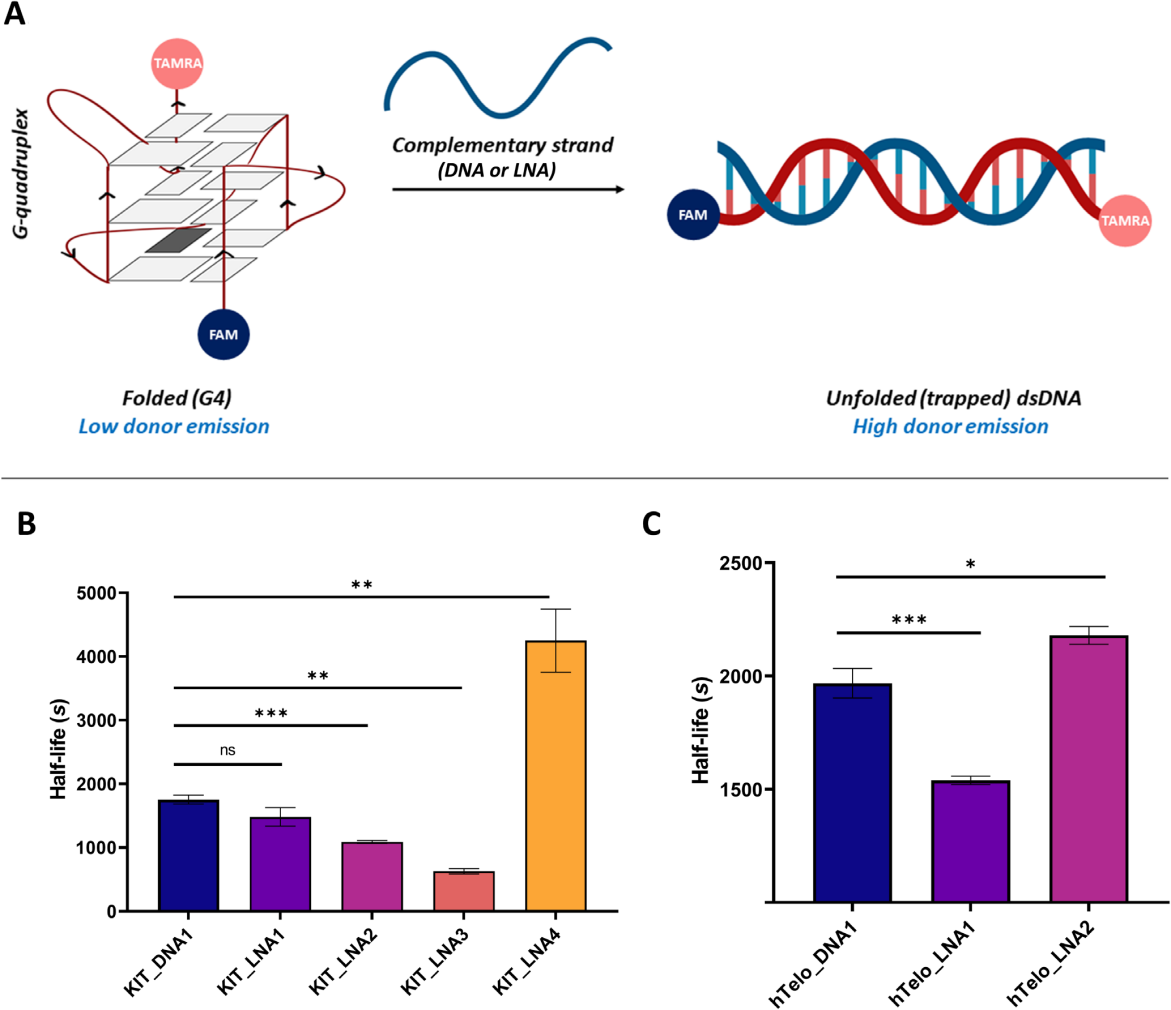
(**A**) Schematic depicting the principles of the FRET G4-disruption assay. c-KIT1 G4 dually labelled with FAM (donor) and TAMRA (acceptor) fluorophores is disrupted in presence of DNA or LNA-modified reverse complementary strand. When the donor fluorophore (FAM) is excited, there is a Föster energy transfer to the acceptor fluorophore (TAMRA), which is sensitive to the distance between the donor and the acceptor. As the G4 gets disrupted to form a duplex with the LNA/DNA probes, the distance between the donor and the acceptor increases, resulting in a concomitant decrease in the energy transfer, which increases donor-emission. (**B**) Half-life of c-KIT1 obtained using the FRET G4-disruption assay when treated with unmodified DNA or KIT_LNA1—4 probes. (**C**) Disruption half-lives of hTelo G4 when treated with full-length DNA (hTelo_DNA1) or LNA (hTelo_LNA1 or hTelo_LNA2). Experiments were performed in three independent replicates with SEM range plotted. *, ** and *** represent statistically significant results at *P*< 0.05, *P*< 0.01 and *P*< 0.001, respectively. ns represents differences that are not statistically significant (*P*> 0.05).

To investigate if different LNA-probes could disrupt c-KIT1 G4 with better efficiency than their DNA analogue, we employed a FRET-based G4-disruption assay previously described in literature (40). This assay uses a dually labelled c-KIT1 sequence consisting of a donor fluorophore (FAM) and an acceptor fluorophore (TAMRA, for sequence see Supplementary Table S1). Briefly, when the FAM is excited, there is an energy transfer to the TAMRA. The energy transfer efficiency is sensitive to the distance between the donor and the acceptor. As the G4 gets disrupted in the presence of the LNA probes to form a duplex, the distance between the donor and the acceptor increases, resulting in a decrease in the energy transfer and a concomitant increase in donor-emission (Figure 2A). Therefore, by monitoring the time-dependent increase in donor emission as the G4 is exposed to the DNA/LNA probes, it is possible to measure kinetics of the disruption process. Assuming a pseudo first-order disruption process as previously described in literature (41), the acquired fluorescence traces can be fit exponentially to obtain a characteristic G4 half-life of the disruption process.

We started our kinetic investigation by measuring the invasion half-life of c-KIT1 G4 when treated with the reverse-complementary DNA probe (**KIT_DNA1**). Since K^+^ is known to have a strong effect on G4-stability (42), we adjusted K^+^ concentrations to have sufficiently slow G4-disruption rates (∼30–60 min for c-KIT1) for accurate kinetics measurements. To achieve this, we performed our assay using 100 nM of labelled c-KIT1 annealed in presence of 5 times molar excess of DNA probe **KIT_DNA1** in a Tris–HCl buffer (pH 7.4) containing 1 mM KCl. We then followed the disruption of the G4 by monitoring FAM emission over time, which yielded a half-life of c-KIT1 G4 of 1753 ± 70 s (Figure 2B, Supplementary Figure S4A and Supplementary Table S3).

Interestingly, when treating c-KIT1 G4 with 5 equivalents of LNA-modified complementary probes under the same conditions, we observed that the G4-disruption rate varied greatly depending on the type of LNA probe used (Figure 2B). Specifically, when the LNA-modifications were positioned opposite to the G-tetrads in **KIT_LNA3**, the half-life of G4-disruption was significantly reduced (628 ± 40 s, *P* = 0.0002), corresponding to a ∼3-fold acceleration of the G4-disruption triggered by **KIT_LNA3** invasion compared to what was measured with the DNA probe (**KIT_DNA1**). Conversely, when LNA modifications were placed in the loops (**KIT_LNA1**), the measured G4 half-life (1481 ± 144 s, *P* = 0.1657) was comparable to the DNA probe **KIT_DNA1**. Consistent with this trend, targeting the 3′ G-tetrad of c-KIT1 G4 with **KIT_LNA2**, also resulted in a significant reduction of the half-life (1087 ± 24 s, *P* = 0.0009). To confirm that targeting of Gs within the G-tetrads was essential to achieve accelerated G4-disruption, we next tested **KIT_LNA4** in which the modifications are scrambled throughout the sequence without exclusively targeting the G-tetrads. Surprisingly, **KIT_LNA4** yielded a significant increase of the G4 half-life (4247 ± 494 s, *P* = 0.0075), which corresponds to a ∼2.5-fold deceleration of the G4-disruption process compared to that with unmodified DNA (**KIT_DNA1**) (Figure 2B, Supplementary Figure S4A-E, Supplementary Table S3). This observation suggests a G4-disruption mechanism whereby the positioning of the LNA modifications within the probe can have dramatic effects on the observed G4-disruption kinetics.

A clear trend that emerges from this initial screening is that LNA modifications, particularly when placed complementary to the guanines involved in G-tetrad formation, can significantly accelerate the rate of c-KIT1 G4-disruption. Collectively, this data revealed a strong position-dependent effect of LNA modification in oligonucleotide probes on the half-life of c-KIT1 G4, which could serve as guiding-principles for rational design of effective G4-disrupting probes.

### LNA-modified probes targeting hTelo confirm a strong positional-dependence of LNA modification on G4-disruption

Based on the striking positional dependence of the G4-disruption kinetics observed for **KIT-LNAs**, we questioned if a similar trend existed among other G4s. To test this, we used the human telomeric G4 sequence (hTelo), which adopts a mixed type topology (43), and designed a subset of LNA-modified probes to target this sequence using the same principles as the c-KIT1 G4 targeting (Table 2).

**Table 2.** List of oligonucleotide sequences used to target hTelo G4. Bold with underline refers to LNA-modified nucleosides

Sequence ID	Sequence (5′ to 3′)
**hTelo_LNA1**	**CCCT** AACCCTAACCCTAACCC
**hTelo_LNA2**	CCC**T**AACC**C**TA**A**CCCT**A**ACCC
**hTelo_DNA1**	CCCTAACCCTAACCCTAACCC

Specifically, **hTelo_LNA1** was designed to target the guanines in the 3′ G-tetrad, and **hTelo_LNA2** contained scrambled LNA modifications throughout the sequence across loops and G-tetrads. We then subjected these hTelo-targeting LNAs to similar conditions as the c-KIT1 G4 in the FRET G4-disruption assay. Briefly, we mixed 100 nM of a FAM/TAMRA dually labelled hTelo oligonucleotide (Supplementary Table S1) in presence of 5 times molar excess of the complementary strands (either DNA or LNA) in a Tris–HCl buffer (pH 7.4) containing 100 mM KCl.

Gratifyingly, we observed a significant reduction in the half-life of hTelo G4 when treated with 5.0 equivalent of **hTelo_LNA1** (1539 ± 18 s, *P* = 0.001), corresponding to a ∼1.5-fold acceleration of G4-disruption compared to unmodified DNA probe **hTelo_DNA1** (1967 ± 65 s) (Figure 2C, Supplementary Figure S5A-B, Supplementary Table S4). In contrast, treatment with the probe **hTelo_LNA2** containing the scrambled LNA modifications across the loops showed a modest increase in the half-life (2179 ± 39 s, *P* = 0.02) relative to the **hTelo_DNA1** control (Figure 2C, Supplementary Figure S5C and Supplementary Table S4). These results are consistent with what was observed on c-KIT1 and corroborate that G4 disrupting-efficiency of probes strongly depends on the position of the LNA within the probe, which can be strategically designed to accelerate (or slow down) G4-disruption in a sequence-specific manner.

### Truncated LNA-modified probes can still efficiently disrupt G4s

We next investigated if the G4-disruption potential of these probes could be retained in shorter sequences truncated around the LNA-modifications. Short LNA-probes are desirable due to their synthetic simplicity and improved cellular uptake, which is crucial for the LNA-based probes to study G4-biology in *in vitro* and *in vivo* models (44). We therefore designed short LNA probes (Table 3) by truncating the full length LNA probes that had shown promising efficiencies of G4-disruption. This generated c-KIT1 or hTelo probes that target only half of the G4-folding sequence (Table 3).

**Table 3.** Truncated short LNA probe designs

Sequence ID	Sequence (5′ to 3′)
**KIT_LNA2**	T**CCCT**CCTCCCAGCGCCCTCCC
**KIT_LNA_short**	T**CCCT**CCTCC
**KIT_DNA_short**	TCCCTCCTCC
**hTelo_LNA1**	**CCCT** AACCCTAACCCTAACCC
**hTelo_LNA_short**	**CCCT** AACCCT
**hTelo_LNA_short2**	C**C**C**T**AA**C**CC**T**
**hTelo_DNA_short**	CCCTAACCCT

**Figure 3.**
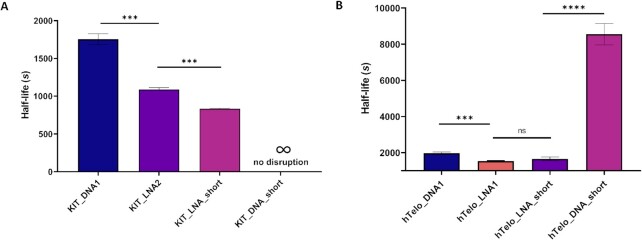
(**A**) Bar plot depicting c-KIT1 G4 half-life measured with short LNA/DNA probes. Treatment of c-KIT1 G4 with **KIT_LNA_short** resulted in a significant reduction in the G4-disruption half-life compared to the full-length analogue **KIT_LNA2**, whereas treatment with **KIT_DNA_short** showed no evidence of G4-disruption (no change in FRET emission over time). (**B**) Treatment of hTelo G4 with the short probe **hTelo_LNA_short** resulted in half-lives comparable to those obtained with the full-length LNA probe (**hTelo_LNA1**). The short DNA probe **hTelo_DNA_short** significantly increased half-lives. Experiments were performed in three independent replicates and plotted with corresponding SEM intervals. **** and *** represent statistical significance at *P*< 0.0001 and *P*< 0.001. ns represents statistically non-significant difference (*P*> 0.05).

To target c-KIT1, we designed **KIT_LNA_short**, which is inspired by **KIT_LNA2** but limited to the first 10 nucleotides in length. Similarly, we designed **hTelo_LNA_short** for targeting the hTelo G4 by truncating **hTelo_LNA1** to the first 10 nucleotides. We then tested these short LNAs for both c-KIT1 and hTelo G4s using the FRET G4-disruption assay. Remarkably, when 100 nM of c-KIT1 in a Tris.HCl buffer at pH 7.4 in 1 mM K^+^ was treated with **KIT_LNA_short**, we were able to observe a significant reduction in half-life (831 ± 8 s, *P* = 0.0002), corresponding to a ∼2-fold acceleration compared to the longer unmodified **KIT_DNA1** (1753 ± 70 s) (Figure 3A, Supplementary Figure S4F and Supplementary Table S3). It is worth noting that **KIT_LNA_short** (831 ± 8 s, *P* = 0.006) is significantly more efficient in accelerating c-KIT1 G4-disruption than its full-length analogue **KIT_LNA2** (1087 ± 24 s), suggesting the potential for an active role of the LNA modifications in disrupting this G4-structure. Conversely, with a truncated probe lacking LNA modifications **KIT_DNA_short**, we failed to observe any G4-disruption, confirming that LNA modifications are essential for G4-disruption. Similarly, for the hTelo system, we found that the short LNA probe **hTelo_LNA_short** under similar conditions (100 nM G4 in a Tris–HCl buffer at pH 7.4 in 100 mM K^+^) showed efficient disruption of the hTelo G4, yielding a lifetime of 1655 ± 105 s, which is comparable (*P* = 0.337) to that observed with the longer LNA-modified analogue **hTelo_LNA1** (1539 ± 18 s), while remarkably faster than the *iso*-sequential DNA control **hTelo_DNA_short** (8551 ± 593 s, *P* = 0.0003) (Figure 3B, S5D–E, Supplementary Table S4). We noticed that the FRET-unfolding trace for **hTelo_DNA_short** displayed an initial fast decay followed by a slow one (Supplementary Figure S5D), which might reflect a more complex unfolding kinetic nature elicited by treatment with this short probe. To consistently apply pseudo-first order model to all dataset, we have limited our analysis to a one-phase decay function reflecting the slow phase to calculate the half-life of **hTelo_DNA_short**, which is more likely to represent the probe invading the G4. To further confirm these effects, we observed that treatment with a truncated probe, **hTelo_LNA_short2**, where the LNA modifications were scrambled, resulted in a half-life of 3014 ± 18s (Supplementary Figure S6 and Supplementary Table S4), which is significantly higher (*P* = 0.002) than **hTelo_LNA_short**, supporting that the position of LNAs in the probe is critical to achieve efficient G4-disruption. These results demonstrate that shorter LNA-modified probes are highly efficient to reduce the disruption half-life of G4s, displaying comparable or better efficiency than their full-length analogues. This key observation suggests a model in which the LNA modifications invade the G4s and promote its disruption independently from the length of the hosting DNA sequence.

To further validate whether these observations may also be true under physiologically relevant ionic conditions, we performed PAGE gel analysis to assess and compare the ability of the short probes **KIT_LNA_short** and **KIT_DNA_short** to disrupt c-KIT1 G4s under higher K^+^ conditions. Briefly, we mixed (25 nM of Cy5-labelled) c-KIT1 G4 in Tris.HCl buffer at pH 7.4 containing 100 mM KCl with a 10-fold excess of the probe (either LNA or DNA), which was then followed by PAGE to separate products based on their molecular weights. The formation of a higher molecular weight species (indicative of LNA-DNA heteroduplex formation) was observed only when the c-KIT1 G4 was treated with **KIT_LNA_short** (Supplementary Figure S7). In contrast, treatment with **KIT_DNA_short** provided no evidence of duplex formation, confirming that for short probes the presence of the LNA-modification is essential for effective G4-disruption. These results were consistent with the electromobility shifts observed when the G4-forming c-KIT1 sequence was annealed (i.e. pre-formed) in the presence of **KIT_LNA_short** (Supplementary Figure S8), confirming that the observed higher molecular weight species can be ascribed to the duplex.

### Single-molecule optical tweezers experiments reveal that short LNAs can deplete mechanical stable G4s that could stall polymerases

**Figure 4.**
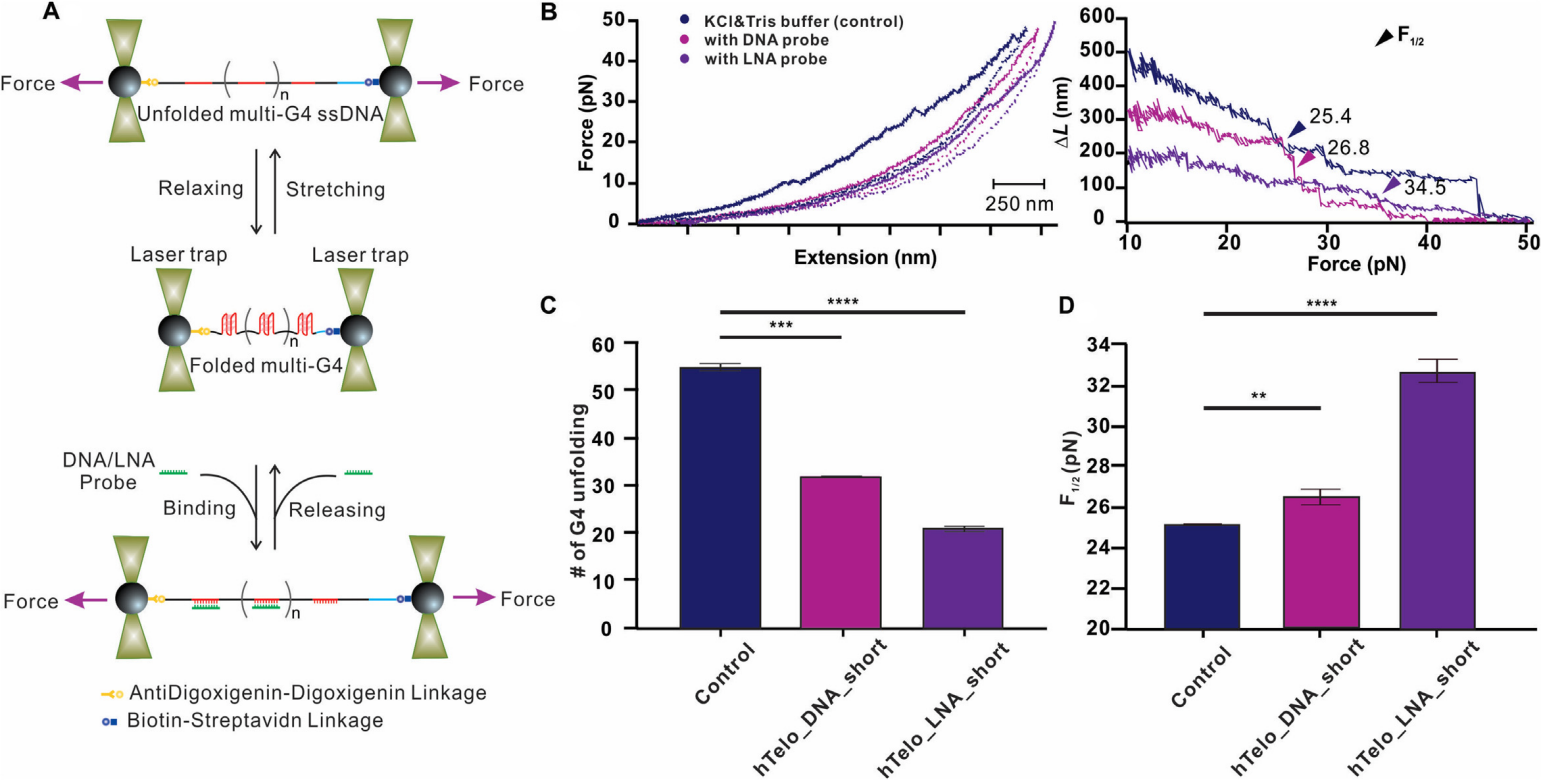
(**A**) Schematic of disrupting an ssDNA that contains multiple units of telomeric G4s without (top) and with (bottom) G4 disrupting DNA or LNA probes. (**B**) Force vs extension curves (left, solid and dotted traces depict stretching and relaxing processes, respectively) and Δ*L* versus force curves (right) for the same molecule in a 10 mM Tris buffer (pH 7.4) supplemented with 100 mM KCl. Blue, buffer only. Pink and purple represent 100 nM hTelo_DNA_short and hTelo_LNA_short, respectively. Arrows indicate *F*_1/2_, where 50% GQ structures were mechanically disrupted. Bar diagrams of (**C**) total number of disrupted GQ and (**D**) *F*_1/2_ values in different solutions. Blue: buffer only; Pink or Purple depict the same buffer added with 100 nM hTelo_DNA_short or hTelo_LNA_short, respectively. Bar charts represent mean values from at least *N*= 5 independent measurements. Error bars represent standard error of mean (SEM). *, ** or *** represent statistical significance at *P*< 0.05, *P*< 0.01 and *P*< 0.001, respectively.

We next investigated if the ability of short LNA-probes to accelerate G4 disruption can be leveraged to disrupt stable G4s that would stall polymerase under physiologically relevant conditions. Since polymerases are motor proteins with a stall force below 20 pN (45), the mechanical stability of G4 becomes a critical factor to evaluate its capability to stall polymerases. Therefore, we performed single-molecule experiments using an optical tweezer platform that directly measures the mechanical stability of G4s and detects disruption upon LNA treatment (Figure 4A). To this end, we first prepared a multi-telomeric G4-forming DNA construct by rolling circle amplification (46) (Supplementary Figure S1A), followed by the comparison of the mechanical disruption events of G4s in this construct in the presence and the absence of LNA/DNA probes (Figure 4B–D). Compared to traditional mechanical disruption experiments where only one G4 is investigated at a time (47), this multi-G4 construct allows to probe the mechanical property of many G4s within a single force-ramping cycle, which drastically increases the throughput. Due to the scenario that many G4s may get disrupted simultaneously when force is increased, rupture events corresponding to disruption of individual G4 units are not obvious. Instead, we observed large hysteresis between disruption and refolding F-X curves (Figure 4B), indicating disruption of many G4s units. At 50 pN, all telomeric G4s are expected to be disrupted, whereas at >10 pN, disrupted G4 will not refold (48). These two force boundaries (10–50 pN) were used to calculate the change in contour length (Δ*L*, see Supporting Information and Supplementary Figure S2, S3 for details) occurred at each disruption force. Since disruption of each telomeric G4 is equivalent to Δ*L* of 9 nm (calculation see Supporting Information (47,48)), such Δ*L* versus *F* plot (Figure 4B) allows us to find total number of G4s formed in the construct (at 10 pN) in presence of DNA or LNA probes. As shown in Figure 4C, we found that treatment with either **hTelo_DNA_short** (32 folded G4s) or **hTelo_LNA_short** (21 folded G4s) decreased the number of folded G4s in the construct (55 G4s in buffer). This confirms what was measured by FRET assays for hTelo, demonstrating that **hTelo_LNA_short** is a more efficient G4-disruptor compared to its DNA analogue (Figure 3B).

We also compared the half force (*F*_1/2_) at which 50% of all telomeric G4s present in the construct are mechanically disrupted under different conditions (Figure 4 B&D). We found that **hTelo_LNA_short** (*F*_1/2_ = 34.5 pN) significantly increased the average mechanical stability of the telomeric G4s compared to the untreated sample (*F*_1/2_ = 25.4 pN) or the **hTelo_DNA_short** sample (*F*_1/2_ = 26.8 pN). This suggests that in the presence of **hTelo_LNA_short**, although formation of G4s was reduced, the surviving G4s had conformations that render increased mechanical stabilities compared to those with or without **hTelo_DNA_short** (Figure 4D). Therefore, our high-throughput platform for simultaneous disruption of multiple G4 units can be potentially used to screen mechanically stronger G4 structures in the presence of G4 destabilizers, which allows us to decipher structural elements responsible for the mechanical property of G4s, as well as to directly assess the mechanical stability of the G4s disrupted by the probes.

### Short LNA probes can restore stalled polymerase progression in G4-containing templates

**Figure 5.**
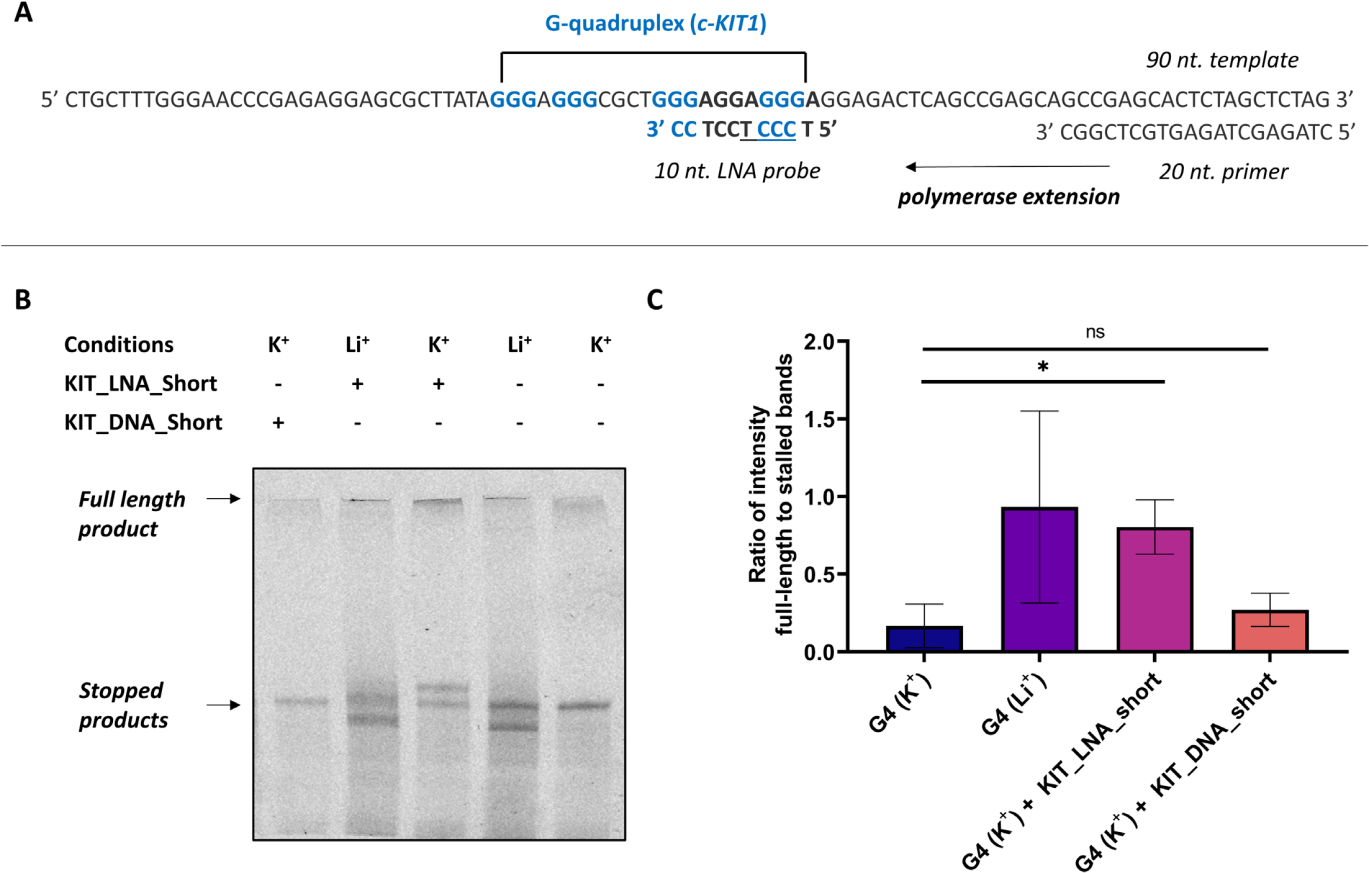
(**A**) Schematic representation of the template and primer used for the single-extension assay. (**B**) Urea-PAGE showing distribution of the full-length and the stalled products when extending the template in the presence or absence of either **KIT_LNA_short** or **KIT_DNA_short** under different conditions. (**C**) Quantified intensity ratio of the full-length *vs* the stalled product band. Higher ratio suggests greater extension efficiency and lower polymerase stalling. Error bars represent standard error of mean (SEM). Means were calculated from *N*= 3 independent experiments. Asterisk * represents statistical significance (*P*< 0.05) whereas ns represents results that are not statistically significant (*P*> 0.05).

We then questioned if the G4-disruption properties displayed by short LNA probes could be leveraged to disrupt G4-formation in a biochemical assay. Specifically, we intended to assess if the ability of LNA-probes to disrupt mechanically stable G4s observed in our single-molecule assay could be exploited to relieve polymerase stalling at G4 sites. To this end, we investigated the capability of **KIT_LNA_short** to aid DNA polymerases to process templates containing c-KIT1 G4. To assess this, we used a well-established single-extension assay (49,50) on a DNA template (∼90 nt, for sequence see Supplementary Table S1) that contains the c-KIT1 G4-forming sequence located 31 nt at the 5′ end. The template was replicated using a Cy5 labelled primer (20 nt, for sequence see Supplementary Table S1), which resulted in the formation of replication products that were analysed by gel electrophoresis (Figure 5A and B) (51).

The template was subjected to single step extension under physiological ionic conditions that are known to favour G4 formation (100 mM K^+^) or do not favour G4 formation (100 mM Li^+^). The polymerase is expected to stall at the c-KIT1 G4 under K^+^ conditions, which leads to truncated extension products resolved in a urea-denaturing gel. Under conditions that do not promote G4-formation (i.e. Li^+^), more efficient formation of the full-length product should be observed. The ratio of the full-length product versus the stalled product can be quantified as the extent of the polymerase progression under different experimental conditions (49,51). As expected, we observed that polymerase progression for the template was ∼6-fold lower when c-KIT1 G4 was formed in K^+^ compared to Li^+^ (Figure 5C and Supplementary Table S5), which indicates G4-dependent stalling of the polymerase at c-KIT1 G4.

When the template annealed in 100 mM K^+^ was treated with **KIT_LNA_short** (1.3 μM), we observed a ∼5-fold increase (*P =*0.04) in polymerase extension compared to the untreated template (Figure 5C and Supplementary Table S5). A clear increase in the intensity of the full-length product band can also be detected (Figure 5C) when the G4 is treated with **KIT_LNA_short**, which is comparable to what measured under Li^+^ conditions. It is worth underlining that such a strong increase in the fully extended products in presence of **KIT_LNA_short** was obtained under 100 mM K^+^, suggesting that the ability of this probe to accelerate c-KIT1 disruption, initially observed at 1 mM KCl in the disruption assays (Figure 3A), can be retained under more physiologically relevant conditions. Along with the increased full-length product formation when treated with the short LNA probe, the appearance of a new higher molecular weight stalled product was also observed, which we ascribed to the DNA/LNA duplex formed upon exposure to the probes that can also cause partial polymerase stalling.

In contrast, when the G4-containing template was treated with DNA probe **KIT_DNA_short** (1.3 μM), we failed to observe any significant (*P* = 0.593) rescue of polymerase progression. These results suggest that short LNA probes can be used to abrogate polymerase-stalling at G4-sites by disrupting G4s under physiologically relevant conditions. These results also indicate that the acceleration in G4-disruption and the depletion of mechanically stable G4s triggered by LNA probes are sufficient to perturb G4-prevalence and stability in a way that can affect biochemical process, such as polymerase elongation. Altogether this further validates the potential for LNA-based probes to be used in cells to investigate G4-biology.

### LNA-modified probes can disrupt c-KIT1 G4 and perturb gene-expression in cells

**Figure 6.**
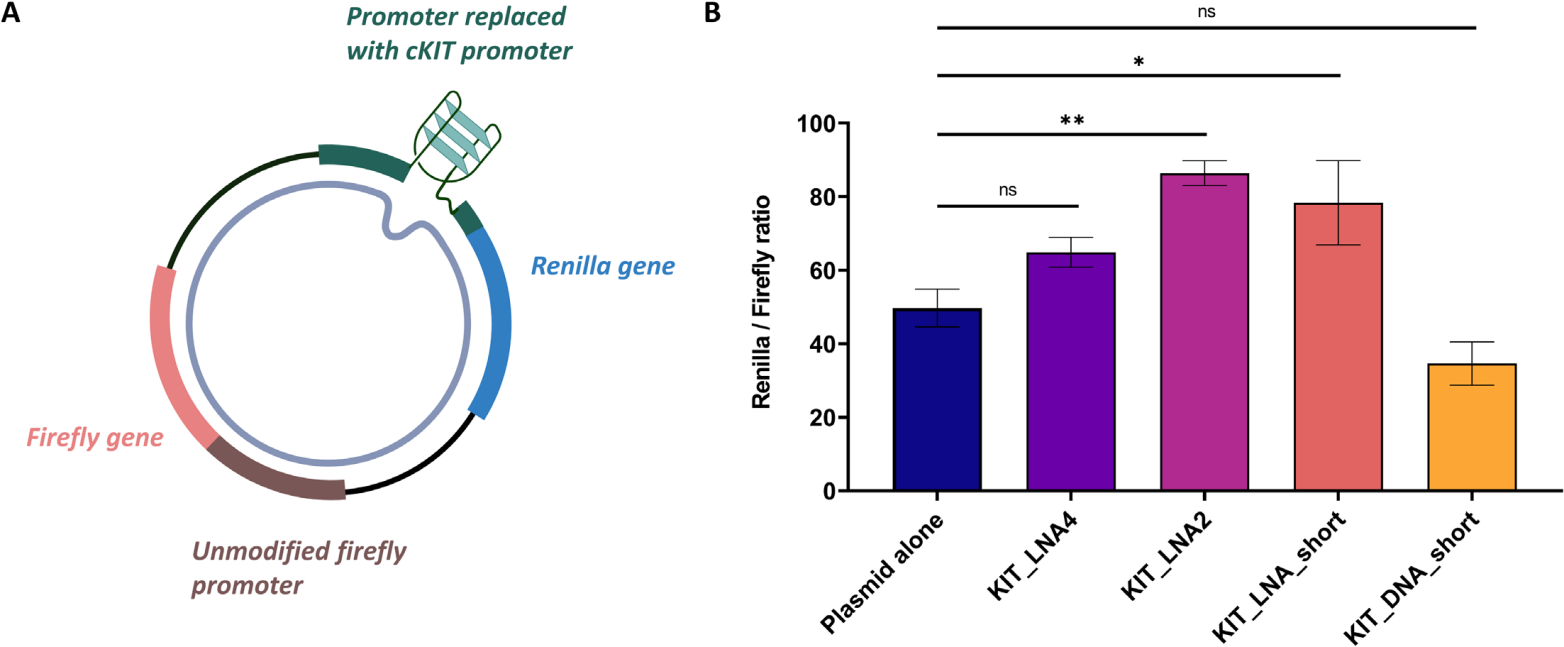
(**A**) The pc-KIT plasmid used in the dual luciferase reporter assay with c-KIT promoter region controlling *Renilla* expression. (**B**) Transfection of the plasmid alone or co-transfection with DNA or LNA probes in HEK293T revealed significant differences in the ability of the probes to perturb relative gene-expressions, as assessed by the ratio of the *Renilla/Firefly* expression levels under the different conditions tested. Error bars represent Standard error of Mean (SEM). Means were computed from *N*= 3 independent biological experiments. Asterisks * and ** represent statistical significance at *P*< 0.05 and *P*< 0.01, respectively, whereas ns represents results that are not statistically significant (*P*> 0.05).

Inspired by the *in vitro* results, we next questioned if the G4-disrupting properties of these LNA-probes could be used to perturb G4-folding in cells. To investigate this, we employed a previously described dual-luciferase reporter system (Figure 6A) (52). Briefly, this assay relies on a plasmid (pc-KIT) that contains two luciferase genes – *Renilla/Firefly*. The luminescence of these two reporter genes can be used to assess their relative expressions. In the pc-KIT plasmid, the expression of Renilla is under the control of the c-KIT promoter containing the c-KIT1 G4 forming sequence, whilst the expression of Firefly is under the control of a non G4-forming promoter sequence (HSV TK promoter). This plasmid was transfected into HEK293T cells in the presence or the absence of the KIT LNA/DNA probes for 24h, followed by the luminescence-based measurement of gene-expressions. Since the promoter that controls Firefly expression does not contain the c-KIT1 G4, we would expect that our probes would only affect the Renilla expression levels, which is controlled by the c-KIT promoter.

Treatment of the transfected cells with **KIT_LNA2** and its shorter version, **KIT_LNA_short**, led to a statistically significant increase in gene expression of the *Renilla* gene when compared to the untreated sample (Figure 6B). Specifically, treatment of the pc-KIT1 plasmid with 25 μM **KIT_LNA2** resulted in an increase in gene expression of ∼2-fold (*P* = 0.009) 24 h after transfection, whereas **KIT_LNA_short** led to an increase in gene expression of ∼1.5-fold (*P* = 0.03) compared to untreated sample. In contrast, **KIT_LNA4** and the control DNA probe **KIT_DNA_short**, which are not expected to efficiently disrupt the G4, failed to provide any significant change in gene-expression relative to the untreated plasmid.

A previously reported study using the same pc-KIT plasmid (52) showed that mutating c-KIT1 sequence to prevent c-KIT1 G4-formation led to an ∼2.5-fold increase in gene expression, which is comparable to what we measured after treatment with **KIT_LNA2**. This further supports a model in which the increase in gene-expression measured in our experiments is a consequence of LNA-mediated disruption of the c-KIT1 G4. Our results suggest that LNA-probes can elicit G4-disruption in a cellular context, indicating a potential link between half-life of G4-disruption and its implication on gene-expression that could be further leveraged to study G4-biology.

## DISCUSSION

Current methods to investigate G4-biology mostly rely on either small-molecule or antibody-based approaches (53,54), which typically work by recognising general features of the structure (i.e. G-tetrads) for stabilisation of the G4. This approach has been highly successful for the detection and mapping of G4s on a genome-wide scale (55). However, it becomes increasingly apparent that molecular probes that can disrupt, rather than stabilise, G4s are equally important to investigate biological processes regulated by these DNA secondary structures. Small-molecules that attain G4-destabilisation have been described (22,25), but their application is limited by two key factors: (i) they often require significant synthetic efforts and they are not currently commercially available and (ii) they would trigger global G4-disruption, preventing the investigation of the biological role of individual G4s. LNA-modified oligonucleotides have been previously identified in literature as promising tools to target and disrupt G4s and have the potential to selectively target individual G4s by base-pair recognition (30). However, to develop LNA-modified G4 disrupting probes that can be applied in cells, a key consideration is how to best position these modifications without creating a DNA/LNA complex too stable to be disassembled in cells. Indeed, the position-dependent effects of LNA are well documented in the antisense oligonucleotide (ASO) design (56), where it has been observed that *iso*-sequential LNA probes vary greatly in their *in vitro* potency depending on their locations. We questioned if a similar positional-effect could be exploited to improve LNA-modified G4-disrupting probes.

Our disruption assays with two topologically different G4s (c-KIT1 and hTelo) demonstrated that LNA-modified probes can not only disrupt but also accelerate the disruption rate of these model G4 systems. Crucially, our data revealed that the position of the LNA modification is key for ‘fine-tuning’ the G4-disrupting abilities. When LNA-modifications were placed complementary to the G-tetrad, we observed significant reductions in the half-life of G4-disruption. Strikingly, when the same number of LNA modifications were distributed in the loops or randomly dispersed, there was a significant increase in the half-life of G4-disruption, suggesting a slow-down of the G4 disruption kinetics. These observations were similar for both G4 systems studied here, suggesting that the design of LNA probes to target and disrupt specific G4s is a generalisable strategy.

To further assess the role of LNA-modifications to promote G4-disruption, we investigated shorter LNA probes of 10 nts in length. These probes target half lengths of the c-KIT1 and hTelo G4-forming sequences. We found they were still able to efficiently induce G4-disruption. By using a FRET-based assay, we demonstrated that the short LNA-modified sequences were able to efficiently disrupt and accelerate the disruption kinetics of G4s with a comparable or higher efficiency than the full-length probes, which further indicates an active role of the LNA-modifications in promoting G4-disruption. Application of such short LNA probes in a single-molecule optical tweezer platform revealed that these LNA probes were able to deplete hTelo G4s. This key observation suggested that LNA probes can abrogate stable G4s, which stall polymerase in replication or transcription bubbles. To test a potential application of such LNA probes to disrupt G4s in biochemical processes, we have employed a single step-extension assay to evaluate whether short LNA-probes could relieve polymerase stalling at G4-sites. Consistent with the single-molecule experiments, this assay demonstrated that short LNA probes were able to disrupt the G4 and increase polymerase extension of templates containing G4s, whilst the equivalent *iso*-sequential short DNA probes showed no such capability.

We then tested these probes in a dual luciferase reporter assay to investigate their ability to perturb transcription in cells. By using a plasmid system that contains the promoter region of the oncogene c-KIT, we revealed that short LNA probes targeting c-KIT1 G4 were able to enhance gene-expression in cells. Strikingly, treatment with the very same sequences lacking LNA modifications did not display any change in gene-expression levels. These results are in agreement with the observed increase in gene-expression measured when formation of c-KIT1 G4 was prevented by sequence mutations in the pc-KIT plasmid (52). Similarly, a small molecule that preferentially disrupts c-KIT1 G4 *in vitro* was also found to be correlated with elevated c-KIT1 expression in cells (22). The increased gene-expression upon treatment of our LNA probes supports a model in which the LNA probes perturb G4 formation in cells. We anticipate our evidence can open up new avenues in the rational design of LNA-based, selective G4-disruptors that would greatly help to study the cell biology of these DNA secondary structures.

## DATA AVAILABILITY

All the data reported in this paper are available in the manuscript or supplementary information. Raw gel and microscopy images can be requested to the corresponding author.

## Supplementary Material

gkac569_Supplemental_File
