# Supplementary material for: Short LNA-modified oligonucleotide probes as efficient disruptors of DNA G-quadruplexes
Source: Nucleic Acids Res. 2022 Jul 8;50(13):7247–59. doi: 10.1093/nar/gkac569 (PMC9303293; doi:10.1093/nar/gkac569)
Supplement: gkac569_Supplemental_File [file gkac569_supplemental_file.pdf]

Supplementary Data

Short LNA-modified oligonucleotide probes as efficient disruptors of DNA G-quadruplexes

Souroprobho Chowdhury[1,2], Jiayi Wang[4], Sabrina Pia Nuccio[1,2], Hanbin Mao[4*] and Marco Di Antonio[1,2,3*]

[1] Imperial College London, Chemistry Department, Molecular Sciences Research Hub, 82 Wood Lane, London W12 0BZ, UK
[2] Institute of Chemical Biology, Molecular Sciences Research Hub, 82 Wood Lane, London W12 0BZ, UK
[3] The Francis Crick Institute, 1 Midland Road, London NW1 1AT, UK
[4] Department of Chemistry and Biochemistry, Kent State University, Kent, OH, USA, 44242.

* To whom correspondence should be addressed. Tel: +44 (0)20 7594 5866; or +1 330 672 9380
Email: m.di-antonio@imperial.ac.uk (MDA) or hmao@kent.edu (HM, for single-molecule studies).

Table S1. G4-forming oligonucleotides used for FRET, PAGE and PCR experiments

Oligonucleotide	Sequence (5' → 3)
**cKIT1 (Cy5)**	Cy5/AGG GAG GGC GCT GGG AGG AGG GGC
**cKIT1 (FAM-TAMRA)**	FAM/AGG GAG GGC GCT GGG AGG AGG GGC/TAMRA
**cKIT1 (PCR template)**	TCT GCT TTG GGA ACC CGA GAG GAG CGC TTA TAG GGA GGG CGC TGG GAG GAG GGA GGA GAC TCA GCC GAG CAG CCG AGC ACT CTA GCT CTA G
**cKIT1 (PCR primer)**	Cy5/CTA GAG CTA GAG TGC TCG GC
**hTelo (FAM-TAMRA)**	FAM/GGG TTA GGG TTA GGG TTA GGG/TAMRA

Table S2. UV melting temperatures for oligonucleotides targeting KIT1 G4-forming sequence.

Oligonucleotide	**Melting Temperature** (Mean ± Standard Error) (*N*=3 independent replicates)
KIT_DNA	72.3 ± 0.6
KIT_LNA1	82.6 ± 0.8
KIT_LNA2	80.6 ± 0.8
KIT_LNA3	84.6 ± 0.8
KIT_LNA4	82.0 ± 0.5

Rolling Circle Amplification (RCA) and chamber design for optical tweezer experiments.

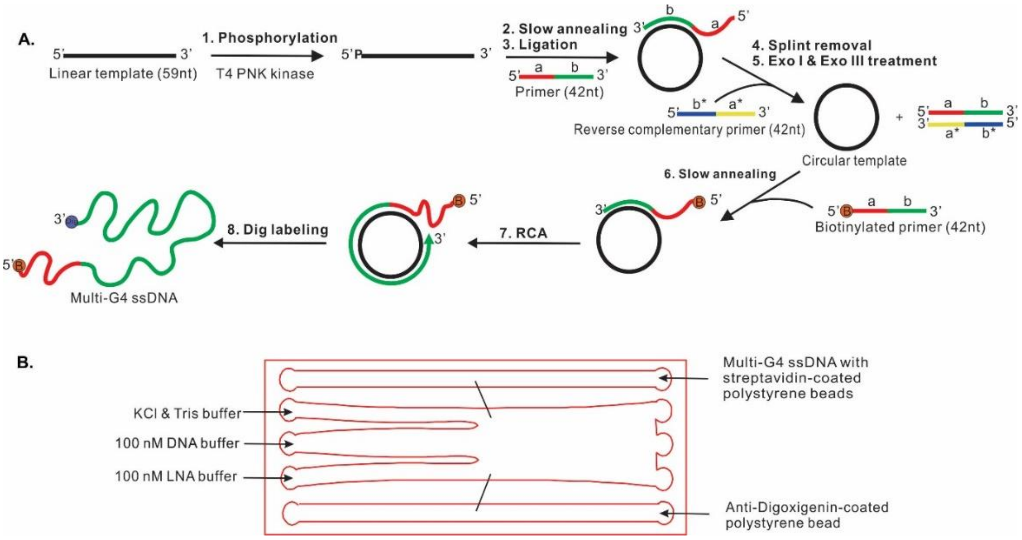

Figure S1. (**A**). Synthesis of an ssDNA construct that contains multiple telomeric G4 units. Dig represents digoxigenin. RCA depicts rolling circle amplification. **(B)** Microfluidic chamber used in the mechanical unfolding experiments.

Construction of ΔL-force curves

As shown in Figure S3, the change in contour length (ΔL) during unfolding of one telomeric G4 unit is given by,

$\Delta L = L\text{-}L_0$ (Eqn S1)

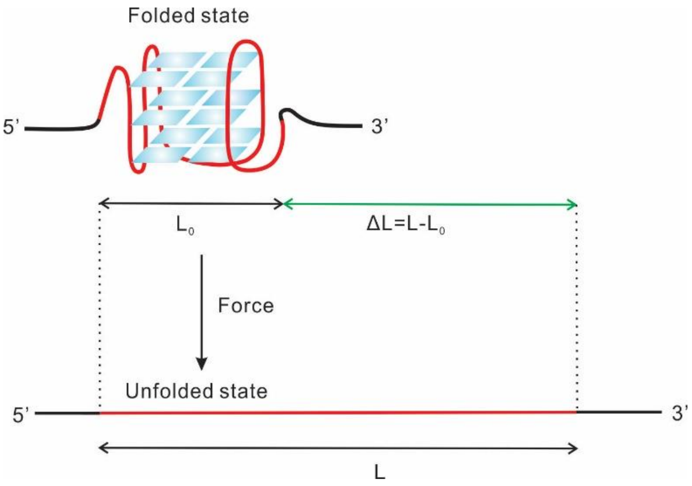

Figure S2. Calculation of the change in contour length (ΔL) during unfolding of a telomeric G4 unit.

where L is the contour length of unfolded G4 and L_0 is the end-to-end distance of the folded G4 (L_0 = 1.1 nm) (1). L is calculated as 9.5 nm by the expression L = n * L_{nt}, in which n represents the number of nucleotides in the folded human telomeric G4 (21 nts) and L_{nt} represents contour length per nucleotide (0.45 nm/nt) (2). Therefore, the expected ΔL during unfolding of a human telomeric G4 is 8.4 nm.

For multi-G4 containing ssDNA construct, the unfolding process may contain simultaneous unfolding of many G4 units. The change in contour length (ΔL) for a multi-G4 construct is calculated using the following equation S2:

$$\Delta x = \Delta L \left[coth\left(\frac{F.b}{k_B T}\right) - \frac{k_B T}{F.b} \right] \left(1 + \frac{F}{S}\right)$$

(Eqn S2)

here b is the Kuhn length (b=1.6 nm) (3), S is the stretch modulus of ssDNA (S=800 pN) (3), and the change in extension (Δx) is calculated as the difference in the extension between the stretching and relaxing curves (see Figure S4, left).

The ΔL vs Force curves (Figure S4 right) were constructed from the force-extension curves (Figure S4 left). At each force (F), ΔL calculated from the Δx using Eqn S2 was plotted against the force to obtain the ΔL-F plot.

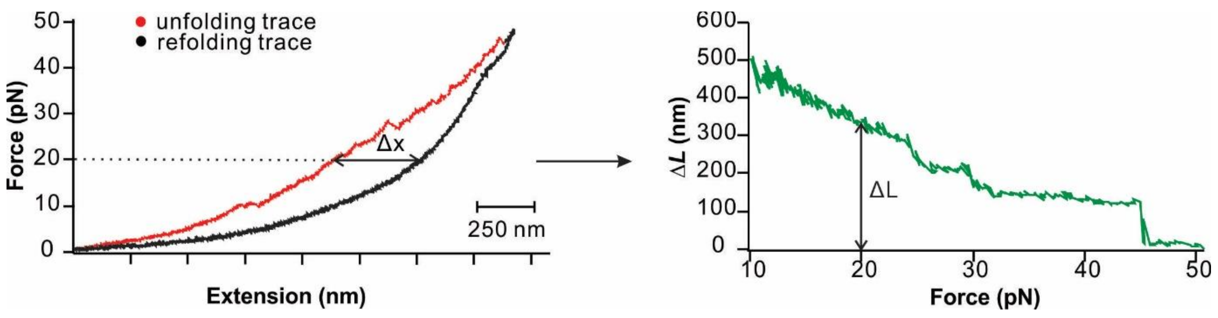

Figure S3. Construction of a ΔL-force curve (right) from a force-extension curve (left). At each force (F), difference in extension (Δx) between the stretching and relaxing force-extension curves (left) is converted to ΔL using eqn S2, which is then plotted again F to obtain the ΔL-F plot (right).

FRET-emission traces for G4-disruption studies

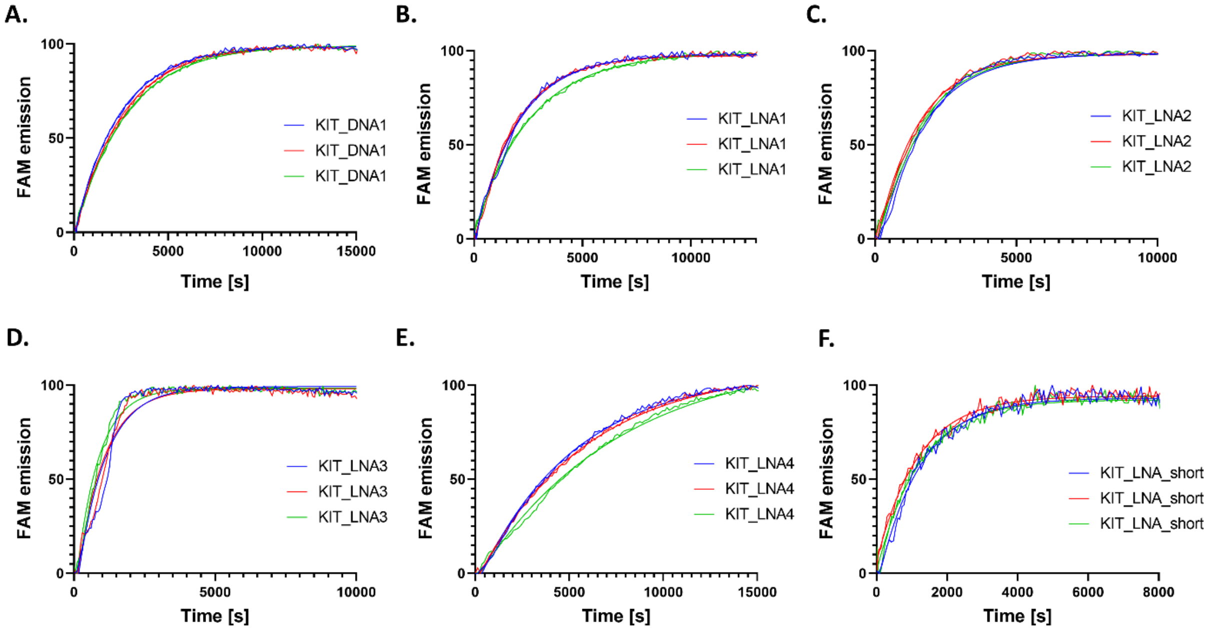

Figure S4. FRET emission traces for G4-disruption assay using a dually labelled cKIT1 G4 system when treated with **(A) KIT_DNA1 (B) KIT_LNA1 (C) KIT_LNA2 (D) KIT_LNA3 (E) KIT_LNA4 (F) KIT_LNA_short**

Table S3. G4 half-lives obtained for cKIT1 by fitting FRET data to one-phase exponential decay functions along with corresponding R squared values for the model.

Molecule ID	Half Life (s)	R squared (Goodness of fit)
**KIT_LNA1**	1373	0.9937
**KIT_LNA1**	1305	0.9967
**KIT_LNA1**	1767	0.9979
**KIT_LNA2**	1112	0.99
**KIT_LNA2**	1038	0.9925
**KIT_LNA2**	1112	0.9913
**KIT_LNA3**	680	0.9431
**KIT_LNA3**	657.5	0.9631
**KIT_LNA3**	547.4	0.9787
**KIT_LNA4**	3568	0.9966
**KIT_LNA4**	3964	0.998
**KIT_LNA4**	5209	0.9958
**KIT_DNA1**	1638	0.9974
**KIT_DNA1**	1740	0.9975
**KIT_DNA1**	1882	0.9979
**KIT_LNA_short**	832.2	0.9727
**KIT_LNA_short**	817.7	0.9817
**KIT_LNA_short**	845.9	0.979

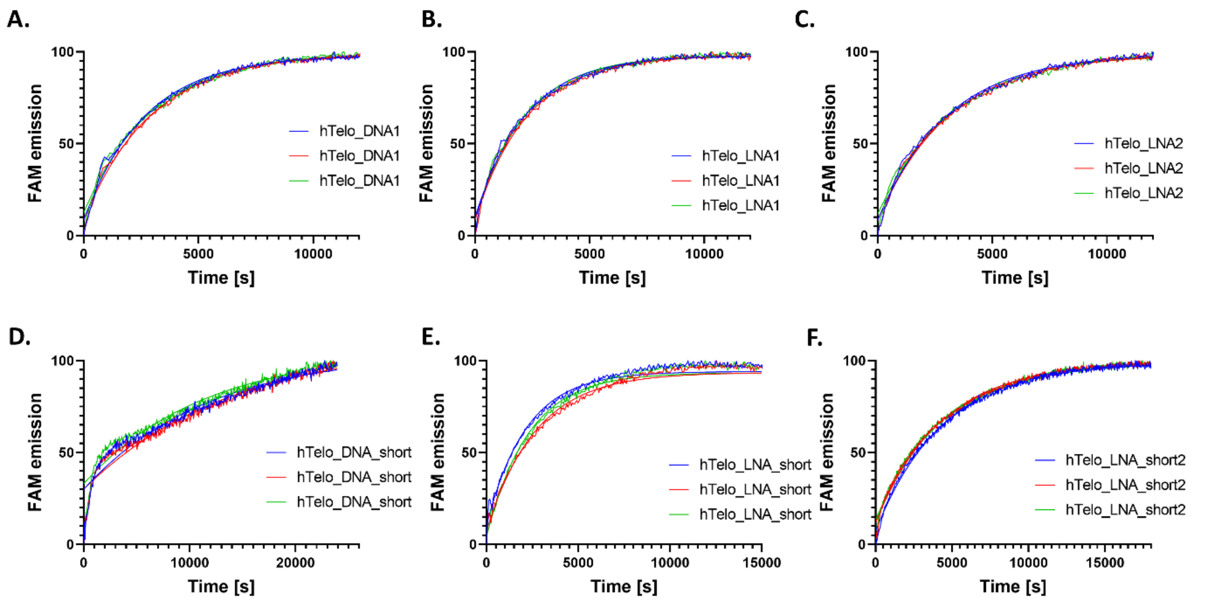

Figure S5. FRET emission traces for G4-disruption assay using a dually labelled hTelo G4 system when treated with (**A**) **hTelo_DNA1** (B) **hTelo_LNA1** (C) **hTelo_LNA2** (D) **hTelo_DNA_short** (E) **hTelo_LNA_short** (**F**) **hTelo_LNA_short2**. The FRET kinetic trace observed for **hTelo_DNA_short** displayed in panel (**D**) revealed a bimodal phase decay characterised by a fast-unfolding step followed by a slow phase. The calculated half-life for **hTelo_DNA_short** reflects the slow phase.

Table S4. G4 disruption half-lives obtained for hTelo by fitting FRET data to one-phase exponential decay functions along with corresponding R squared values for the model.

Molecule ID	Half Life (s)	R squared (Goodness of fit)
**hTelo_DNA1**	1837	0.9929
**hTelo_DNA1**	2035	0.9958
**hTelo_DNA1**	2031	0.9921
**hTelo_LNA1**	1513	0.9941
**hTelo_LNA1**	1575	0.9942
**hTelo_LNA1**	1530	0.9936
**hTelo_LNA2**	2120	0.9934
**hTelo_LNA2**	2163	0.9947
**hTelo_LNA2**	2255	0.9942
**hTelo_DNA_short**	8082	0.9509
**hTelo_DNA_short**	9730	0.9578
**hTelo_DNA_short**	7842	0.9475
**hTelo_LNA_short**	1509	0.9454
**hTelo_LNA_short**	1860	0.965
**hTelo_LNA_short**	1598	0.9515
**hTelo_LNA_short2**	3046	0.9964
**hTelo_LNA_short2**	2984	0.9958
**hTelo_LNA_short2**	3013	0.9951

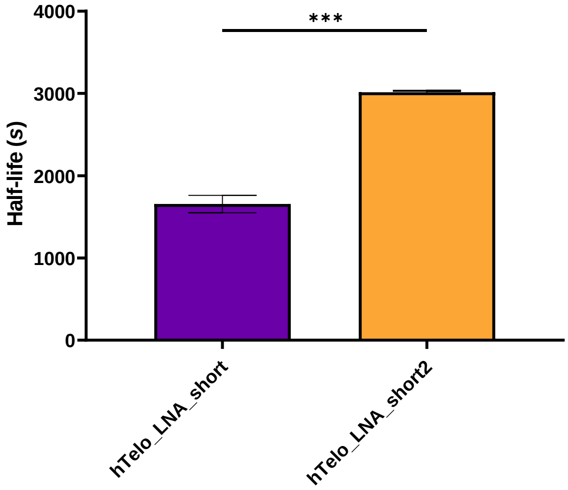

Figure S6. Half-life of G4-disruption for hTelo in 100 mM K^+ buffer obtained using FRET assay when treated with 5 equivalents of **hTelo_LNA_short** (purple) or of **hTelo_LNA_short2** (yellow). Data are represented as means obtained from 3 independent experiments. Error bars represent the Standard Error of Mean (SEM).

Native PAGE analysis of cKIT1 targeting with LNA or DNA.

Method

500 nM of 5' Cy5 labelled cKIT-1 oligonucleotide sequence (Cy5/AGG GAG GGC GCT GGG AGG AGG GGC) was annealed in 100 mM KCl, 10 mM Tris-HCl pH 7.4 buffer or in 100 mM LiCl, 10 mM Tris-HCl pH 7.4. Reactions were performed in the relevant buffer (either K^+ or Li^+) to a final volume of 10 μL, containing 25 nM of the labelled oligonucleotide (0.5 μL added from the 500 nM annealed stock), and 5-fold excess of the LNA probe or the DNA probe (250 nM, 2.5 μL added from a 1 μM stock), which was incubated at 25 ºC for 6 hours (Figure S1). For experiments with positive control (Figure S2, Lanes 3 and 5), the pre-formed duplex were formed by annealing the Cy5 labelled cKIT-1 sequence in 100 mM KCl, 10 mM Tris-HCl pH 7.4 buffer at 500 nM, in presence of 5-fold excess (2.5 μM) of either KIT_LNA_short or KIT_DNA_short probes. The 6% PAGE gel was prepared (4.4 mL 2X TBE, 3.5 mL 30% acrylamide, 140 μL of 10% APS, 18 μL of TEMED, and 9.3 μL of miliQ water) and pre-run at 100V for 15 mins in 0.5X TBE buffer. After incubation, 2 μL of 50% glycerol was added to each reaction before mixing and loading to each lane and the gel was run at 100V for 25 mins at room temperature. The imaging of the gels was performed using Typhoon FLA 9500 (GE Healthcare).

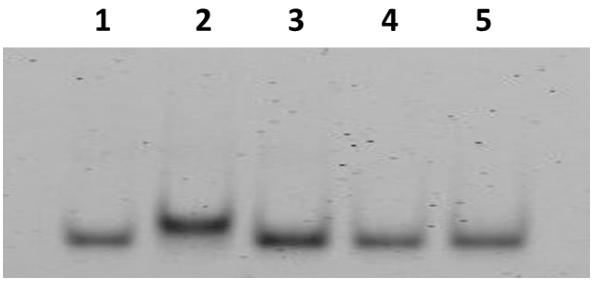

Figure S7. Polyacrylamide gel to probe cKIT1 disruption under physiological ionic conditions using either **KIT_LNA_short** or **KIT_DNA_short** probes.

Lane 1: G4 in 100 mM K^+

Lane 2: G4 in 100 mM K^+ incubated with **KIT_LNA_short**

Lane 3: G4 in 100 mM Li^+

Lane 4: G4 in 100 mM K^+ incubated with **KIT_DNA_short**

Lane 5: G4 in 100 mM Li^+ incubated with **KIT_DNA_short**

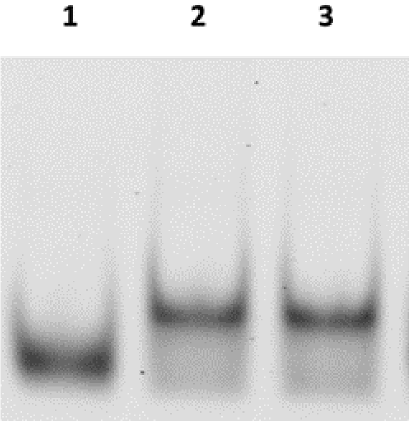

Figure S8. Polyacrylamide gel (PAGE) analysis of cKIT1 G4-forming sequence labelled with Cy5 in 100 mM K^+ buffer. Lane 1: cKIT1 G4 alone, Lane 2: cKIT1 treated with 5 equiv. **KIT_LNA_short**, Lane 3: cKIT1 pre-annealed with 5 equiv. of **KIT_LNA_short** to pre-form the duplex.

Table S5. Intensity ratios obtained from polymerase stalling assay using gel-quantification.

Conditions (3 replicates for each)	Intensity ratio of full-length band to stopped product band
G4 (K^+)	0.032057996
	0.449069
	0.021584
G4 (Li^+)	0.120644715
	2.145595
	0.529145
G4 (K^+) + KIT_LNA_short	0.807154805
	1.10605
	0.498936
G4 (Li^+) + KIT_LNA_short	0.307888619
	0.617764
	0.264227
G4 (K^+) + KIT_DNA_short	0.18622521
	0.48398
	0.141816
G4 (Li^+) + KIT_DNA_short	0.243567871
	0.446012
	0.356457

References

1. Koirala,D., Mashimo,T., Sannohe,Y., Yu,Z., Mao,H. and Sugiyama,H. (2006) Intramolecular folding in three tandem guanine repeats of human telomeric DNA. *Chem. Commun*, **48**, 2006-2008

2. Yu,Z., Gaerig,V., Cui,Y., Kang,H., Gokhale,V., Zhao,Y., Hurley,L.H. and Mao,H. (2012) Tertiary DNA structure in the single-stranded hTERT promoter fragment unfolds and refolds by parallel pathways via cooperative or sequential events. *J. Am. Chem. Soc.*, **134**, 5157–5164.

3. Smith,S.B., Cui,Y. and Bustamante,C. (1996) Overstretching B-DNA: The Elastic Response of Individual Double-Stranded and Single-Stranded DNA Molecules. *Science*, **271**, 795–799.
